# Dispositional mindfulness, interoceptive awareness, and panic-agoraphobic spectrum, in a general population sample

**DOI:** 10.1017/S1092852925000100

**Published:** 2025-02-10

**Authors:** Mario Miniati, Alessandra Battani, Laura Palagini, Rebecca Ciacchini, Ciro Conversano, Graziella Orrù, Giulio Perugi, Donatella Marazziti, Angelo Gemignani

**Affiliations:** 1 Psychiatric Clinic, Department of Clinical and Experimental Medicine, University of Pisa, 56126 Pisa, Italy; 2 Department of Surgical, Medical and Molecular Pathology and of Critical Care Medicine, University of Pisa, 56126 Pisa, Italy

**Keywords:** dispositional mindfulness, awareness, interoception, panic-agoraphobic spectrum, subthreshold

## Abstract

**Objective:**

To investigate dispositional mindfulness (DM), interoceptive awareness (AI), and the occurrence of panic-agoraphobic spectrum signs and symptoms in a non-clinical population.

**Methods:**

The study involved a general population sample (*n* = 141), aged between 18 and 40, evaluated with the Panic-Agoraphobic Spectrum Self-Report Lifetime Version (PAS-SR-LT), the Mindful Attention Awareness Scale (MAAS), and the Multidimensional Assessment of Interoceptive Awareness (MAIA). Instruments were administered with an online procedure (Microsoft Forms). The Bioethics Committee of the University of Pisa approved the study (protocol #0105635/2023).

**Results:**

Panic-agoraphobic spectrum was detected in more than 50% of our sample (PAS-SR Total Score ≥ 35). According to the MAIA assessment, subjects who scored above the PAS-SR threshold were more afraid and less able to distract attention from their bodily sensations. A binary logistic regression analysis was performed to evaluate if MAIA and MAAS dimensions were able to predict the presence of a more severe panic-spectrum symptomatology. The PAS-SR cut-off score <35 versus ≥35 was adopted as the dependent variable. “Age” and “gender” (categorical), MAAS, and MAIA scores were inserted as covariates. MAAS “Total Score” (OR = .955; CI = .924–.988; *p* = .007), and MAIA “Not worrying” (OR = .826; CI = .707–.964; *p* = .016) predicted for a less relevant panic-agoraphobic spectrum phenomenology, resulting as “protective” factors.

**Conclusions:**

Progression from interoceptive processing to mindful abilities to resilience against panic catastrophizing of bodily sensation is far from being clarified. However, our study provides information on a panic-agoraphobic spectrum phenotype characterized by low levels of mindful attitudes and less interoceptive abilities.

## Introduction

Panic attacks and agoraphobia are frequent in the general population.^1^ Signs and symptoms of panic might run underdiagnosed, mainly because of their heterogeneity in the clinical presentation.^2^ ^–^^4^ Different subtypes of panic disorder (PD) have been described on the basis of the observed predominant symptomatology. The first classification of PD included two subtypes, namely a “*respiratory subtype*”, characterized by physical respiratory/cardiovascular manifestations, and a “*cognitive symptoms subtype*”, in which subjective distress and fear were predominant.^5^ However, this classification has been criticized and considered as partially reliable. According to a different approach, patients with PD might show a number of phenotypes because of the occurrence of manifestations not included in the list of PD typical signs and symptoms: the so-called *“panic-agoraphobic spectrum”* manifestations surrounding the psychopathological “*core”* of the disorder.^6^ ^–^^9^ The panic-agoraphobic spectrum encompasses atypical panic symptoms, as well as sub-syndromal conditions (not reaching the threshold for a full-blown PD) that might represent subtle manifestations of an illness diathesis in the general population or might become the expression of sub-threshold comorbidity for patients with psychiatric disorders other than PD.^10^ The “*spectrum model*” was conceived to include subthreshold, isolated, atypical, early onset, and residual symptoms, or “*trait-like*” characteristics that can enhance vulnerability to a specific psychopathological area.^2^ In order to make a measurable panic-agoraphobic spectrum, a structured clinical interview (SCI-PAS) and the corresponding self-report questionnaire (PAS-SR) were validated. Both instruments consisted of 114 items and ten factors, as described in detail elsewhere.^2^

It is well-known that, independently from the clinical or theoretical approach to this psychopathological area, a relevant number of PD manifestations belong to the realm of the so-called “*mind-body connection*”.^11^ ^–^^19^ Given that, mindfulness-based interventions, supporting time-limited states of mindfulness, have been proposed as treatments for subjects with panic symptoms.^15^ ^–^^19^ However, the response was rather heterogeneous across studies for a number of reasons, including the different “*dispositional mindfulness*” (DM) of involved subjects.^20^

Dispositional Mindfulness (DM) is the “*subjective inclination to be mindful*’, namely the “*natural inclination*” to pay attention (on purpose and non-judgmentally) to the present moment.^20^ ^–^^22^

The observant attitude of DM could provide a clear picture of the outer (visual, auditive, tactile, etc.) and inner experiences (bodily sensations, thoughts, emotions, etc.), a better understanding of emotional states, needs, and values, an enhancement of empathy abilities, a greater self-knowledge, a stronger self-regulation, and a more efficient interoceptive awareness (IA).^22^ Interoceptive awareness (IA) is a complex dimension whose definition is still a matter of debate, as it does not exclusively refer to physiological sensations” perception and includes appraisals, beliefs, past experiences, expectations, and emotions related to bodily perceptions.^23^ ^–^^31^ Several authors claimed that paying attention to bodily sensations, especially those related to stress and anxiety, would enhance the feeling of distress.^12^ ^,^^23^ However, according to the mindfulness approach, the act of paying attention to the body might have opposite effects, depending on its attitude.^20^ ^,^^23^ ^,^^26^

The attention dedicated to bodily sensations might determine a misinterpretation (“*bodily sensations are threats to be scared of and to avoid”)* when accompanied by hypervigilance and catastrophic automatic thoughts.^12^ ^,^^14^ ^,^^20^ ^,^^23^ ^,^^26^ ^,^^28^ ^,^^32^ ^,^^33^

Conversely, if attention is shaped as an observant, open, and non-judgmental attitude, focusing on bodily sensations might mitigate anxiety levels and lower the risk for panic symptoms.^14^ ^,^^34^

However, the potential relationship between DM, IA, and proneness to panic symptoms is far from being clarified. Our study aimed to evaluate whether different levels of both DM and IA might influence the occurrence of panic-agoraphobic spectrum manifestations in a general population sample. We hypothesized that both lower DM and IA levels might predispose to the presence of panic-agoraphobic spectrum dimensions. On the other hand, DM could be a protective factor against panic-agoraphobic spectrum manifestations, especially when IA is good.

## Methods

### Participants and procedures

This present study was cross-sectional, observational, and non-profit. One hundred and forty-one community volunteers were recruited between September and October 2023, utilizing an online procedure. Subjects who agreed to participate read the information on the protection of personal data before completing the online questionnaires and provided their consent to the assessment. Obtained data from the online procedure (Microsoft Form®) were automatically transformed into codes. Participants” anonymization was ensured, according to the European norms on privacy and data protection, at the very moment of accessing the questionnaires. To be included in the study, participants had to be aged between 18 and 40 years, with no severe physical illness or current psychiatric disorder, including substance, alcohol use/abuse, or suicidal ideation. The Bioethics Committee of the University of Pisa approved the study (protocol # 0105635/2023). The authors assert that all procedures contributing to this work comply with the ethical standards of the relevant national and institutional committees on human experimentation and with the Helsinki Declaration of 1975, as revised in 2008.

### Questionnaires

We collected and analyzed data on demographic characteristics, including age, gender, relationship status, working activity, education, and area of residence (urban/suburban/rural). Moreover, we utilized three self-assessment instruments described below, namely the Panic-Agoraphobic Spectrum Self-Report, Lifetime Version (PAS-SR-LT)^2^, the Mindful Attention Awareness Scale (MAAS),^21^ and the Multidimensional Assessment of Interoceptive Awareness (MAIA).^34^

### Panic-agoraphobic spectrum self-report, lifetime version (PAS-SR-LT)^2^

The PAS-SR-LT consists of 114 items, rated as “present” (YES) or “absent” (NO). A classical exploratory factor analysis, based on a tetrachoric correlation matrix and oblique rotation, extracted 10 factors accounting overall the 66.3% of the variance of the questionnaire: (1) panic symptoms; (2) agoraphobia; (3) claustrophobia; (4) separation anxiety; (5) fear of losing control; (6) drug sensitivity and phobia; (7) medical reassurance; (8) rescue objects; (9) loss sensitivity; (10) reassurance from family members.

In two studies, the clinical threshold of ≥35 items was adopted to identify subjects with a higher lifetime burden of panic-agoraphobic spectrum symptoms, and in a third one, the instrument demonstrated its cross-cultural validity.^35^ ^–^^37^

In our study, the instrument showed a very good internal consistency (Cronbach’s *α* = 0.941).

### *Mindful attention awareness scale (MAAS)* ***^21^***

The MAAS is commonly utilized to assess individual mindfulness disposition. It consists of 15 items evaluating the subjective inclination to pay attention on purpose and nonjudgmentally to the present moment. The items are rated on a scale from 1 to 6 (1 = almost always, 2 = very frequently, 3 = somewhat frequently, 4 = somewhat infrequently, 5 = very infrequently, 6 = almost never). Higher scores suggest a stronger subjective tendency to be receptive to both inner and outer present experiences. MAAS showed good internal consistency in our study (Cronbach’s *α* = 0.908).

### *Multidimensional assessment of interoceptive awareness (MAIA)* ***^29^*** ^,^ ***^34^***

The MAIA consists of 32 items rated on a scale from “0” (“Never”) to “5” (“Always”), assessing eight dimensions of interoceptive awareness: (1) ‘Noticing”, on the ability to identify and correctly defining body sensations (uncomfortable, comfortable and neutral ones); (2) “Not-Distracting”, on the tendency to ignore/distract from sensations of pain/discomfort; (3) “Not-Worrying” on the ability of not being distressed and worried when discomfort or pain sensation arise; (4) “Attention Regulation”, on the ability to control the attention towards bodily sensations; (5) “Emotional Awareness”, on the awareness of connections between emotional states and bodily sensations; (6) “Self-regulation” on the ability to regulate psychological distress by paying attentions to bodily sensations; (7) “Body Listening” on the tendency to listen to the body as a source of insight; (8) “Trusting” on the tendency to experience the body as safe and trustworthy. MAIA allows researchers to analyze different psychological aspects of the perception and evaluation of bodily sensations and to differentiate adaptive and maladaptive interoceptive awareness processes. In our study, the scale showed a very good internal consistency (Cronbach’s *α* = 0.920).

### Statistical analyses

Quantitative variables were described by means and/or medians and standard deviations and/or interquartile ranges. Qualitative variables were expressed with frequencies and percentages. The Shapiro–Wilk and the Kolmogorov–Smirnov tests were applied to evaluate whether the variables studied had a normal distribution. For the same purposes, means, medians, distribution, and kurtosis were also assessed. The ANOVA test was used to compare the values of variables with the Gaussian distribution. In the case of non-Gaussian variables, the Kruskal-Wallis test was applied. Any differences or associations between nominal variables were evaluated with the Chi-square test (χ^2^) or Fisher’s exact test, depending on the frequencies detected. Differences between values were analyzed with the *T*-Test for paired data when appropriate; for non-Gaussians variables, the Wilcoxon test was performed. A linear generalized multivariate model (ANCOVA) adjusted for the variable “age” was applied when appropriate. Correlation analyses were applied using both Spearman’s and Pearson’s tests, depending on the distribution of the variables considered. The predictive validity of mindful attitudes and traits on the occurrence of panic-agoraphobic spectrum signs and symptoms was assessed with binary logistic regression analysis, with “PAS-SR Threshold” as the dependent variable and MAIA/MAAS factors scores as independent variables. The level of statistical significance was associated with *p* < .05. Analyses were carried out using IBM SPSS Statistics.

## Results

### Overview

The overall sample consisted of 141 participants, aged between 18 and 40 years (mean age = 25.7 ± 4.9), 116 females (82.2%) (mean age = 25.6 ± 5.1), and 24 males (17.0%) (mean age = 26.6 ± 4.8) and one subject (0.7%) who did not declare the gender (age = 26.0).

According to the Kolmogorov–Smirnov test, the “age” distribution was not normal (*p* = .0001). According to the *U*-Mann–Whitney Test for independent samples, no statistically significant differences were detected in the distribution of the variable “age” between the two samples with gender declared (*p* = .45).

The demographic characteristics of the sample are summarized in Table 1.

**Table 1.** Sociodemographic Information of the Overall Sample and by Gender

	Overall Sample *N* = 141	Males *N* = 24^a^	Females *N* = 116^a^
Age^b^	Mean/SD	Mean/SD	Mean/SD
	25.7 ± 4.9	26.6 ± 4.8	25.6 ± 5.1
Relationship Status	*n*/%	*n*/%	*n*/%
Single	112 (79.4)	20 (83.3)	92 (79.3)
With a Partner	28 (19.9)	4 (16.7)	24 (20.7)
Working activity	*n*/%	*n*/%	*n*/%
Unemployed	4 (2.8)	2 (8.3)	2 (1.7)
Student	99 (70.2)	16 (66.6)	83 (71.5)
Farmer/Worker	3 (2.1)	–	3 (2.6)
Employee	25 (17.7)	4 (16.6)	21 (18.1)
Freelance	5 (3.5)	1 (4.1)	4 (3.4)
Manager	1 (0.7)	1 (4.1)	–
Education	*n*/%	*n*/%	*n*/%
Middle school	5 (3.5)	–	5 (4.3)
High school	50 (52.7)	7 (29.1)	43 (37.0)
Degree	73 (51.7)	13 (54.1)	60 (51.7)
Residency/Ph.D.	12 (8.5)	4 (16.6)	8 (6.9)
Area of residence	*n*/%	*n*/%	*n*/%
Rural	20 (14.1)	4 (16.6)	16 (13.7)
Sub-urban	34 (24.1)	8 (33.3)	26 (22.4)
Urban	86 (60.9)	12 (50.0)	74 (63.8)

a One subject is not displayed; gender not declared.

b Test *U* Mann–Whitney for independent sample: *p* = .45.

### Scales” scores

Scores of the administered scales in the overall sample and by gender are summarized in Table 2. The scores distribution was normal for all instruments” factors and dimensions, except for PAS-SR “Factor 3: Claustrophobia” (*p* = .0001); “Factor 5: Fear of losing control” (*p* = .002); “Factor 7: Medical Reassurance” (*p* = .0001); “Factor 8: Rescue Objects” (*p* = .0001); “Factor 9: Loss Sensitivity” (*p* = .0001); “Factor 10: Reassurance from Family Members” (*p* = .0001) (One-Sample Kolmogorov–Smirnov Test). Accordingly, a non-parametric test was applied for the above-mentioned factors (*U* Mann–Whitney Test for Independent Samples). No statistically significant differences were found between genders, except for the mean scores of the MAIA “Noticing” (*p* = .02), which was significantly higher in the female subjects. Moreover, no statistically significant differences emerged for the distribution of subjects reaching the PAS-SR clinical threshold ≥35 (50.0% in males versus 54.4% in females; df = 1; χ^2^ = .435).

**Table 2.** Scores of the Administered Scales in the Overall Sample and by Gender^c^

	Overall Sample*N* = 141	Males*N* = 24^c^	Females*N* = 116^c^	p
	Mean/SD	Mean/SD	Mean/SD	
MAIA				
*1. Noticing* ^a^	9.6 ± 4.0	7.8 ± 3.7	9.9 ± 4.0	.02
*2. Not-distracting^a^*	6.3 ± 2.6	6.2 ± 2.5	6.4 ± 2.7	.72
*3. Not-worrying^a^*	5.7 ± 2.8	6.0 ± 2.8	5.6 ± 2.9	.46
*4. Attention Regulation^a^*	15.0 ± 7.0	15.8 ± 7.6	14.9 ± 6.9	.56
*5. Emotional Awareness^a^*	12.9 ± 5.1	12.7 ± 5.3	12.9 ± 5.0	.81
*6. Self-regulation^a^*	7.8 ± 3.9	7.8 ± 4.7	7.8 ± 3.8	.96
*7. Body Listening^a^*	5.9 ± 3.1	5.4 ± 3.0	5.9 ± 3.1	.43
*8. Trusting^a^*	6.5 ± 3.5	6.4 ± 3.6	6.5 ± 3.6	.88
MAIA Total Score^a^	69.9 ± 19.2	68.5 ± 19.8	70.3 ± 19.9	.678
MAAS Total Score^a^	62.3 ± 14.8	61.2 ± 14.0	62.7 ± 14.9	.64
PAS-SR Total Score^a^	38.4 ± 17.4	34.1 ± 17.0	39.0 ± 17.2	.20
*Panic symptoms^a^*	9.4 ± 4.6	8.5 ± 4.6	9.6 ± 4.6	.28
*Agoraphobia^a^*	5.3 ± 3.6	4.0 ± 3.6	5.5 ± 3.5	.06
*Claustrophobia^b^*	2.3 ± 2.2	1.9 ± 2.3	2.3 ± 2.2	.28
*Separation Anxiety^a^*	5.0 ± 2.7	4.4 ± 2.6	5.1 ± 2.7	.25
*Fear of losing control^b^*	4.1 ± 2.9	3.7 ± 3.0	4.2 ± 2.8	.43
*Drug sensitivity and phobia^a^*	3.4 ± 2.1	3.4 ± 2.3	3.4 ± 2.1	.98
*Medical Reassurance^b^*	1.2 ± 1.5	1.2 ± 1.4	1.6 ± 1.5	.69
*Rescue Objects^b^*	1.1 ± 1.0	0.9 ± 1.0	1.1 ± 1.1	.27
*Loss Sensitivity^b^*	1.6 ± 1.0	1.6 ± 1.2	1.6 ± 1.0	.87
*Reassurance from family members^b^*	2.0 ± 0.9	1.9 ± 0.9	2.1 ± 0.8	.30
	*n*/%	*n*/%	*n*/%	χ^2^
PAS-SR Total Score <35	65 (46.0)	12 (50.0)	53 (45.6)	.435
PAS-SR Total Score ≥35	76 (54.0)	12 (50.0)	63 (54.4)	

a Student *T*-Test for independent samples.

b Test *U* Mann–Whitney for independent samples.

c One subject is not displayed in the comparison between males and females: gender not declared.

### MAIA and MAAS scores in subjects with PAS-SR score <35 versus PAS-SR score ≥35

We compared MAIA and MAAS scores in subjects who fulfilled the clinical threshold for a panic-agoraphobic spectrum disorder, according to PAS-SR scores (≥35) (53.5%; *n* = 75) versus subjects who scored <35 (46.5%; *n* = 65), performing a Student *T*-Test for independent samples, giving that, as already tested, all considered variables had a normal distribution. Subjects with PAS-SR ≥ 35, scored significantly lower than subjects with PAS-SR < 35 at MAAS Total Score (57.8 ± 13.7 versus 67.9 ± 14.1; *p* = .0001), showing a less represented mindful attitude, and at MAIA “Noticing” (8.5 ± 4.2 versus 10.4 ± 3.6), “Not-Distracting” (6.9 ± 6.8 versus 5.8 ± 2.3; *p* = .019), “Not-Worrying” (in which higher scores are representative of less preoccupation in presence of physical discomfort) (6.7 ± 2.7 versus 4.8 ± 2.7; *p* = .0001), “Emotional Awareness” (11.8 ± 5.5 versus 13.8 ± 4.5; *p* = .023), and “Trusting” (7.3 ± 3.6 versus 5.8 ± 3.3; *p* = .015) domains, as summarized in Table 3.

**Table 3.** MAIA and MAAS Scores in Subjects with PAS-SR Score < 35 Versus PAS-SR Score ≥ 35

	Overall Sample*N* = 141	PAS-SR < 35*N* = 65	PAS-SR ≥ 35*N* = 76	*p* ^a^
	Mean/SD	Mean/SD	Mean/SD	
MAIA				
Noticing	9.6 ± 4.0	8.5 ± 4.2	10.5 ± 3.6	.005
Not-distracting	6.3 ± 2.6	6.9 ± 2.8	5.9 ± 2.3	.019
Not-worrying	5.7 ± 2.8	6.7 ± 2.7	4.7 ± 2.7	.0001
Attention regulation	15.0 ± 7.0	14.6 ± 7.3	15.3 ± 6.9	.573
Emotional awareness	12.9 ± 5.1	11.8 ± 5.5	13.8 ± 4.5	.020
Self-regulation	7.8 ± 3.9	8.2 ± 4.2	7.5 ± 3.7	.266
Body listening	5.9 ± 3.1	5.7 ± 3.2	6.0 ± 3.0	.538
Trusting	6.5 ± 3.5	7.3 ± 3.6	5.8 ± 3.3	.018
MAIA Total Score	69.9 ± 19.2	70.1 ± 20.8	69.8 ± 17.8	.947
MAAS Total Score	62.3 ± 14.8	67.9 ± 14.1	57.5 ± 13.9	.0001
MAAS Average Score (Total score/15)	4.1 ± 0.9	4.5 ± 0.9	3.8 ± 0.9	.0001

a Student *T*-Test for independent samples.

### MAAS threshold: Higher versus lower mindful traits/attitudes

We calculated not only the MAAS total score but also a mean of the 15 items, namely, the “*MAAS Average Score” (MAAS-AS) (total score/15).* As already noticed, higher scores reflect higher levels of DM and lower negative emotional states. In literature, average scores for undergraduate students have been reported to be equal to 3.85; conversely, Zen meditators scored an average of ≥4.38 (Brown et al., 2011). Therefore, we utilized the above-mentioned cut-off to compare subjects with “*higher levels of MA*” (H-MAAS) versus those with “*lower levels of MA*” (L-MAAS). MAAS-AS showed a normal distribution in the overall sample (4.1 ± 0.9; *p* = .201; range: 1.2–6.0) according to the Kolmogorov–Smirnov Test. Seventy-six subjects scored <4.38 (53.9%), and 65 scored ≥4.38 (46.1%).

The two samples showed no differences for age distribution (26.8 ± 5.4 versus 24.9 ± 4.1, respectively; *p* = .140; *U* Mann–Whitney Test for independent samples), nor for gender distribution (41.7% in males versus 47.4% in females; df = 1; χ^2^=.607).

We compared PAS-SR and MAIA domains/factors scores in H-MAAS subjects (≥4.38) versus L-MAAS subjects (<4.38), as summarized in Table 4. Subjects with H-MAAS, namely with high levels of mindful traits and attitudes, scored higher at almost all MAIA domains, except for “noticing” (9.7 ± 4.1 versus 9.5 ± 3.9; *p* = .729) and “emotional awareness” (12.8 ± 5.2 versus 13.0 ± 5.0; *p* = .899).

**Table 4.** MAIA and PAS-SR Scores in Subjects with Low MAAS Average Scores (<4.38) Versus High MAAS Average Scores (≥4.38)

	MAAS-ASLow (<4.38)*n* = 76	MAAS-ASHigh (≥4.38)*n* = 65	*p*
	Mean/SD	Mean/SD	
MAIA			
1. Noticing	9.5 ± 3.9	9.7 ± 4.1	.729
2. Not-distracting	5.9 ± 2.4	6.9 ± 2.8	.022
3. Not-worrying	5.1 ± 2.8	6.3 ± 2.8	.011
4. Attention Regulation	13.9 ± 6.7	16.3 ± 7.2	.042
5. Emotional Awareness	13.0 ± 5.0	12.8 ± 5.2	.899
6. Self-regulation	7.0 ± 3.6	8.8 ± 4.1	.007
7. Body Listening	5.3 ± 3.0	6.5 ± 3.1	.027
8. Trusting	5.4 ± 3.4	7.8 ± 3.2	.0001
MAIA Total Score	65.2 ± 18.9	75.5 ± 18.2	.001
PAS-SR Total Score *^a^*	45.7 ± 17.4	30.0 ± 13.0	.0001
1. Panic Symptoms *^a^*	11.1 ± 4.4	7.5 ± 3.9	.0001
2. Agoraphobia *^a^*	6.4 ± 3.8	4.0 ± 2.9	.0001
3. Claustrophobia *^b^*	2.8 ± 2.3	1.7 ± 1.9	.001
4. Separation Anxiety *^a^*	6.0 ± 2.8	3.9 ± 2.1	.0001
5. Fear of losing control *^b^*	5.4 ± 2.7	2.6 ± 2.3	.0001
6. Drug Sensitivity/Phobia *^a^*	3.7 ± 2.2	3.0 ± 2.0	.052
7. Medical Reassurance *^b^*	1.6 ± 1.7	0.6 ± 1.1	.0001
8. Rescue Objects *^b^*	1.3 ± 1.0	0.9 ± 1.0	.025
9. Loss Sensitivity *^b^*	1.8 ± 1.1	1.3 ± 0.9	.009
10. Reassurance from family members *^b^*	2.2 ± 0.9	2.1 ± 0.9	.253

a Student *T*-Test for independent samples.

b Mann–Whitney *U* Test (2 samples).

More importantly, subjects with H-MAAS reported scores significantly lower in almost all factors of PAS-SR than subjects with L-MAAS, except for Factor 6 “Drug Sensitivity/phobia” (3.0 ± 2.0 versus 3.7 ± 2.2; *p* = .052), and Factor 10 “Reassurance from Family Members (2.1 ± 0.9 versus 2.2 ± 0.9; *p* = .253).

### Correlation analyses

Correlation analyses between MAIA, MAAS dimensions, and PAS-SR domains were performed with the Pearson-r correlation coefficient for PAS-SR normally distributed variables (PAS-SR “Total Score’, “Panic Symptoms’, “Agoraphobia’, “Separation Anxiety, “Drug Sensitivity and Phobia”), and with the Spearman rs correlation coefficient, for not normally distributed variables (PAS-SR “Claustrophobia’, “Fear of Losing Control’, “Medical Reassurance’, “Rescue Objects’, “Loss Sensitivity” and “Reassurance from Family Members”), as summarized in Table 5.

**Table 5.** Correlation Analyses Between PAS-SR Factors and MAAS, MAIA Total Scores and Domains in the Overall Sample (*n* = 141)

	MAAS Total Score	MAIANoticing	MAIA Not distracting	MAIA Not worrying	MAIAAttentionRegulation	MAIAEmotionalAwareness	MAIASelfRegulation	MAIABodyListening	MAIATrusting	MAIATotal Score
PAS-SR Total score *^a^*	−476**	.254**	−.178*	−.389**	−.139	−.203*	−.208*	−.058	−.296**	−.135
PAS-SR Panic Symptoms *^a^*	−.365**	.254**	−.093	−.309**	−.166*	.192*	−.195*	−.038	−.256**	−.110
PAS-SR Agoraphobia *^a^*	−.292**	.327**	−.157	−.341**	−.008	.222**	−.115	.072	−.245**	−.006
PAS-SR Claustrophobia *^b^*	−.382**	.043	−.158	−.185*	−.044	.100	−.137	−.124	−.231**	−.137
PAS-SR Separation Anxiety *^a^*	−.382**	.154	−.068	−.359**	−.074	.147	−.109	.048	−.060	−.045
PAS-SR Fear of losing control *^b^*	−.482**	−.254**	−.119	−.212*	−.136	.130	−.197*	−.117	−.340**	−.139
PAS-SR Drug Sensitivity/Phobia *^a^*	−.169*	.155	−.104	−.395**	−.132	.104	−.092	.028	−.098	−.095
PAS-SR Medical Reassurance *^b^*	−.390**	−.027	−.076	−.260**	−.311**	−.020	−.273**	−.227**	−.220**	−.313**
PAS-SR Rescue Objects *^b^*	−.223**	.165	−.121	−.087	.070	.162	−.108	−.085	−.132	.007
PAS-SR Loss Sensitivity *^b^*	−.302**	−.016	−.015	−.209*	−.159	.033	−.230**	−.169*	−.242**	−.200**
PAS-SR Reassurance from family *^b^*	.156	−.201*	.081	−.038	−.168*	−.154	−.010	−.017	−.079	−.109

**p < .01 (two-tailed);* *p* < .05.

^a^ = Pearson correlations.

^b^ = Rho Spearman correlations.

Table 6 summarized correlations between MAIA and MAAS dimensions. Statistically significant negative correlations were observed between MAAS “Total Score” and all PAS-SR factors (except for “Reassurance from Family Members’; *r* = .156; *p* = ns). namely, PAS-SR “Total Score” (*r* = −.476; *p* < .01), “Panic Symptoms” (*r* = −.365; *p* < .01), “Agoraphobia” (*r* = −.292; *p* < .01), “Claustrophobia” (rs = −.382; *p* < .01), “Separation Anxiety” (*r* = −.382; *p* < .01), “Fear of Losing Control” (rs = −.482; *p* < .01), “Drug Sensitivity and Phobia” (*r* = −.169; *p* < .05), “Medical Reassurance” (rs = −.390; *p* < .01), “Rescue Objects” (rs = −.223; *p* < .01), and “Loss Sensitivity” (rs = −.302; *p* < .01). According to these analyses, when dispositional mindfulness scores get higher, panic-agoraphobic spectrum scores get lower, and vice-versa.

**Table 6.** Correlation Analyses Between MAAS and MAIA Dimensions in the Overall Sample (*n* = 141)

	MAIA Noticing	MAIANot Distracting	MAIANot Worrying	MAIAAttention Regulation	MAIAEmotional Awareness	MAIASelf-Regulation	MAIABody Listening	MAIA Trusting	MAIATotal Score
MAAS Total Score	.070	.175^*^	.175^*^	.270^**^	.015	**.364** ^**^	**.304** ^**^	**.416** ^**^	**.370** ^**^
MAIA Noticing	–	−.178*	−.315**	.299**	.514**	.357**	.524**	.233**	.585**
MAIA Not Distracting	–	–	.132	−.218**	−.121	−.231**	−.117	.068	**−.045**
MAIA Not Worrying	–	–	–	.080	−.168*	.060	−.105	.039	**.090**
MAIA Attention Regulation	–	–	–	–	.408**	.487**	.441**	.417**	.770**
MAIA Emotional Awareness	–	–	–	–	–	.383**	.492**	.244**	.685**
MAIA Self-Regulation	–	–	–	–	–	–	.702**	.525**	.750**
MAIA Body Listening	–	–	–	–	–	–	–	.546**	.780**
MAIA Trusting	–	–	–	–	–	–	–	–	.666**

** *p* < .01 (two-tailed);* *p* < .05, Pearson Correlations.

Correlations between PAS-SR factors and MAIA dimensions were more heterogeneous.

The MAIA “Noticing” was positively correlated with PAS-SR “Total Score” (*r* = .254; *p* < .01), “Panic Symptoms” (*r* = .254; *p* < .01), “Agoraphobia” (*r* = .327; *p* < .01), and negatively with “Fear of Losing Control” (rs = −.254; *p* < .01), and “Reassurance from Family Members” (rs = −.201; *p* < .05).

The MAIA “Not-Distracting” was negatively correlated only with PAS-SR “Total score” (*r* = −.178; *p* < .05).

The MAIA “Not-Worrying” was negatively correlated with PAS-SR “Total Score” (*r* = −.389; *p* < .01), “Panic Symptoms” (*r* = −.309; *p* < .01), “Agoraphobia” (*r* = −.341; *p* < .01), “Claustrophobia” (rs = −.185; *p* < .05), “Separation Anxiety” (*r* = −.359; *p* < .01), “Fear of Losing Control” (rs = −.212; *p* < .05), “Drug Sensitivity and Phobia” (*r* = −.395; *p* < .01), “Medical Reassurance” (rs = −.260; *p* < .01), and “Loss Sensitivity” (*r* = −.209; *p* < .05).

The MAIA “Attention Regulation” was negatively correlated with the PAS-SR “Total Score” (*r* = −.166; *p* < .05), “Medical Reassurance” (rs = −.311; *p* < .01), and with “Reassurance from Family Members” (rs = −.168; *p* < .05).

The MAIA “Emotional Awareness” was negatively correlated with PAS-SR “Total Score” (*r* = −.203; *p* < .05) and positively with “Panic Symptoms” (*r* = .192; *p* < .05) and “Agoraphobia” (*r* = .222; *p* < .01).

The MAIA “Self-Regulation” was negatively correlated with PAS-SR “Total Score” (*r* = −.208; *p* < .05), “Panic Symptoms” (*r* = −.195; *p* < .05), “Fear of Losing Control” (rs = −.197; *p* < .05), “Medical Reassurance” (rs = −.273; *p* < .01), and “Loss Sensitivity” (rs = −.230; *p* < .01).

The MAIA “Body Listening” was negatively correlated with PAS-SR “Medical Reassurance” (rs = −.227; *p* < .01) and “Loss Sensitivity” (rs = −.169; *p* < .05).

The MAIA “Trusting” was negatively correlated with PAS-SR “Total Score” (*r* = −.296; *p* < .01), “Claustrophobia” (*r* = −.256; *p* < .01), “Agoraphobia” (*r* = −.245; *p* < .01), “Claustrophobia” (rs = −.231; *p* < .01), “Fear of Losing Control” (rs = −.340; *p* < .01), “Medical Reassurance” (rs = −.220; *p* < .01), and “Loss Sensitivity” (rs = −.242; *p* < .01).

The MAIA “Total Score” was negatively correlated with “Medical Reassurance” (rs = −.313; *p* < .01) and with “Loss Sensitivity” (rs = −.200; *p* < .01).

Table 6 summarized correlations between MAAS Total Score and MAIA total score/domains. The MAAS total score was positively correlated with MAIA “Not Distracting” (*r* = .175; *p* < .05), MAIA “Not Worrying” (*r* = .175; *p* < .05), MAIA “Attention Regulation” (*r* = .270; *p* < .01), MAIA “Self-Regulation” (*r* = .364; *p* < .01), MAIA “Body Listening” (*r* = .304; *p* < .01), MAIA “Trusting” (*r* = .416; *p* < .01), and with MAIA total score (*r* = .370; *p* < .01). No significant correlations emerged between MAAS “Total Score” and MAIA “Noticing” (*r* = .070; p > .05), or MAIA “Emotional Awareness” (*r* = .015; *p* > .05).

### *Binary logistic regression analysis of subjects with PAS-SR total score <35 versus* ≥ ***35***

A binary logistic regression analysis was performed with the aim of evaluating if MAIA and MAAS dimensions were able to predict the presence of more severe panic-spectrum symptomatology in our general population sample. The PAS-SR cut-off score <35 versus ≥35 was adopted as the dependent variable. “Age” and “gender” (categorical), MAAS, and MAIA scores were inserted in the model as covariates. The MAAS total score (OR = .955; CI = .924–.988; *p* = .007) and MAIA “Not worrying” (OR = .826; CI = .707–.964; *p* = .016) were the only two variables in the model predicting a less relevant panic-agoraphobic spectrum phenomenology, resulting as “protective” factors, as summarized in Table 7.

**Table 7.** Binary Logistic Regression Analysis with PAS-SR ≥ 35 Versus PAS-SR < 35 as Dependent Variable and “Age’, “Gender’, MAAS Total Score and MAIA Dimensions as Co-Variates

	B	E.S.	Wald	df	Sig.	Exp (B)	95% CI for Exp (B)
							Inf. Sup.
MAAS Total Score	−.046	.017	7.233	1	.007	.955	.924 .988
MAIA Noticing	.092	.069	1.758	1	.185	1.096	.957 1.255
MAIA Not distracting	−.092	.088	1.089	1	.297	.912	.767 1.084
MAIA Not Worrying	−.191	.079	5.845	1	.016	.826	.707 .964
MAIA Attention Regulation	.050	.040	1.597	1	.206	1.051	.973 1.137
MAIA Emotional Awareness	.047	.053	.763	1	.383	1.048	.944 1.163
MAIA Self-Regulation	−.106	.086	1.509	1	.219	.900	.760 1.065
MAIA Body listening	.114	.114	1.006	1	.316	1.121	.897 1.402
MAIA Trusting	−.147	.078	3.578	1	.059	.863	.741 1.005
Age	−.052	.045	1.338	1	.247	.950	.870 1.037
Gender	.080	.527	.023	1	.879	1.083	.385 3.046

## Discussion

Relationships between DM, IA, and panic-agoraphobic spectrum dimensions seem to clearly emerge from our study. Panic-agoraphobic spectrum was detected in more than 50% of our sample (PAS-SR Total Score ≥ 35). According to the MAIA assessment, subjects who scored above the PAS-SR threshold (≥35) were more focused on or preoccupied with their physical sensations when compared with subjects who scored <35, even in the absence of a full-blown disorder. Moreover, they were less able to distract attention from their bodily sensations and more aware/less trusting of their body, in line with previous observations.^38^ ^–^^40^

According to correlation analyses, the two MAIA dimensions that were more negatively correlated with almost all PAS-SR factors were “Not worrying” and “Trusting’. Subjects with more proneness to report on panic-agoraphobic spectrum phenomenology were more preoccupied with and less confident about their bodily sensations. Interestingly, in our sample, subjects who scored higher on PAS-SR were also the most aware of the relationships between emotional states and bodily sensations. These findings are in line with the theoretical model of “*catastrophizing interoceptive signals”* as one of the main characteristics of PD.^24^

Panic-agoraphobic physical manifestations are strictly related to the fear of losing control; as a consequence, patients with PD are sensitive and hypervigilant towards interoceptive signals, considered ambiguous and often unpleasant.^39^ ^–^^42^ Additionally, when counterphobic measures are more represented (see, for example, the search for family and medical reassurance), the awareness of different bodily sensations is less efficacious.^43^

Unfortunately, our analyses and the lifetime assessment of PAS-SR did not allow us to define if panic-agoraphobic spectrum signs were primarily present, determining a subsequent/secondary change in the evaluation of bodily sensations or vice versa. Conversely, we can reasonably assume that a pre-existent valid “*mindful attitude”* could be considered one of the “*protective factors*” against panic spectrum signs and symptoms. Therefore, the subjects of our sample with a high mindful attitude (MAAS ≥ 4.38), when compared with those with MAAS < 4.38, scored significantly lower in almost all PAS-SR factors. A higher inclination to pay attention to the present moment on purpose and nonjudgmentally is defining subjects with less panic manifestations, in line with previous observations.^12^ ^,^^28^ ^,^^44^ Moreover, a higher DM in our sample correlated with higher levels of IA. Correlation analyses between MAAS and MAIA scores showed that being aware of presenting was positively correlated with the ability to control one’s attention towards bodily sensations (“Attention Regulation”), with a greater ability to regulate psychological distress (“Self-Regulation”), with a greater tendency to listen to one’s body as a source of insight (“Body Listening”) and with a higher feeling of one’s body as reliable (“Trusting”).^20^ ^,^^30^ ^,^^45^

The relevance of the potential relationships in terms of prediction, between interoception, mindful attitude, and the occurrence of panic-agoraphobic signs and symptoms has been confirmed by the binary logistic regression analysis Interestingly, MAAS total score and MAIA “Not worrying” were the only two variables in the model predicting for a less relevant panic-agoraphobic spectrum phenomenology, both resulting as “protective” factors. We could interpret this finding postulating a hypothetical progression from good levels of interoceptive awareness to good levels of mindful attitude to the less occurrence of panic signs and symptoms.

Progression from interoceptive processing to mindful abilities to resilience against panic catastrophizing of bodily sensation is far from being clarified. However, our study provides information on a panic-agoraphobic spectrum phenotype characterized by low levels of mindful attitudes and less efficacious interoceptive abilities and vice versa.

Our study has several limitations, such as a cross-sectional design not allowing the definition of a cause-effect relationship between the observed dimensions. The questionnaires administered were all online self-reports; therefore, the study might have suffered from participants” inaccuracy in reporting their experiences (recall bias). Lastly, as already noticed in previous studies, it is not easy to operationalize mindfulness in “*discrete items”*: MAAS measures the attentional dimensions of mindfulness, but it does not adequately measure intentional acceptance, curiosity, and kindness qualities.^46^ ^–^^48^

Future studies should also involve a clinical sample of patients with full-blown PD in order to provide information on differences between panic-agoraphobic manifestations, mindful attitudes, and interoceptive characteristics in non-clinical versus clinical populations.
